# Craniofacial divergence by distinct prenatal growth patterns in Fgfr2 mutant mice

**DOI:** 10.1186/1471-213X-14-8

**Published:** 2014-02-28

**Authors:** Susan M Motch Perrine, Theodore M Cole, Neus Martínez-Abadías, Kristina Aldridge, Ethylin Wang Jabs, Joan T Richtsmeier

**Affiliations:** 1 Department of Anthropology, Pennsylvania State University, University Park, PA 16802, USA; 2 Department of Basic Medical Science, University of Missouri-Kansas City School of Medicine, Kansas City, MO 64110, USA; 3 Department of Pathology & Anatomical Sciences, University of Missouri School of Medicine, Columbia, MO 65212, USA; 4 Department of Genetics and Genomic Sciences, Icahn School of Medicine at Mount Sinai, New York, NY 10029, USA; 5 Current address: CRG, Center for Genomic Regulation, Dr. Aiguader, 88, 08003 Barcelona, Spain; 6 Center for Functional Anatomy and Evolution, Johns Hopkins University School of Medicine, Baltimore, MD 21228, USA

**Keywords:** Fibroblast growth factor receptor signaling, Cranial development, Skull growth, Suture, Craniosynostosis, Apert syndrome

## Abstract

**Background:**

Differences in cranial morphology arise due to changes in fundamental cell processes like migration, proliferation, differentiation and cell death driven by genetic programs. Signaling between fibroblast growth factors (FGFs) and their receptors (FGFRs) affect these processes during head development and mutations in FGFRs result in congenital diseases including FGFR-related craniosynostosis syndromes. Current research in model organisms focuses primarily on how these mutations change cell function local to sutures under the hypothesis that prematurely closing cranial sutures contribute to skull dysmorphogenesis. Though these studies have provided fundamentally important information contributing to the understanding of craniosynostosis conditions, knowledge of changes in cell function local to the sutures leave change in overall three-dimensional cranial morphology largely unexplained. Here we investigate growth of the skull in two inbred mouse models each carrying one of two gain-of-function mutations in FGFR2 on neighboring amino acids (S252W and P253R) that in humans cause Apert syndrome, one of the most severe FGFR-related craniosynostosis syndromes. We examine late embryonic skull development and suture patency in *Fgfr2* Apert syndrome mice between embryonic day 17.5 and birth and quantify the effects of these mutations on 3D skull morphology, suture patency and growth.

**Results:**

We show in mice what studies in humans can only infer: specific cranial growth deviations occur prenatally and worsen with time in organisms carrying these *FGFR2* mutations. We demonstrate that: 1) distinct skull morphologies of each mutation group are established by E17.5; 2) cranial suture patency patterns differ between mice carrying these mutations and their unaffected littermates; 3) the prenatal skull grows differently in each mutation group; and 4) unique *Fgfr2*-related cranial morphologies are exacerbated by late embryonic growth patterns.

**Conclusions:**

Our analysis of mutation-driven changes in cranial growth provides a previously missing piece of knowledge necessary for explaining variation in emergent cranial morphologies and may ultimately be helpful in managing human cases carrying these same mutations. This information is critical to the understanding of craniofacial development, disease and evolution and may contribute to the evaluation of incipient therapeutic strategies.

## Background

Growth of the skull is a complex process that combines genetic and environmental information to establish and mineralize the individual bony elements that come together to protect and support the rapidly expanding soft tissues of the head, such as the brain [1]. The well-ordered activity of specific transcription factors regulated by a range of developmental regulatory signals marks the differentiation of osteoblast lineage cells during the ossification process [2]. Among those signaling systems that pattern growth and assembly of the skull are fibroblast growth factors (FGF) and their receptors (FGFRs) whose essential role during development is largely known through the study of missense mutations that cause congenital skeletal diseases including craniosynostosis, achondrodysplasia, and syndromes with dysregulated phosphate metabolism [3,4]. Apert syndrome [OMIM 101200] is caused by gain-of-function mutations in FGFR2, over 99% of which are amino acid substitutions in Ser252Trp (S252W) or Pro253Arg (P253R) [5-7]. Apert syndrome is characterized by premature fusion of cranial sutures (craniosynostosis), midfacial retrusion, and additional complex cranial, neural, limb, and visceral malformations with cognitive ability varying widely, from normal to severely deficient [8]. FGFR2 is widely expressed throughout development: therefore changes in the development of many tissues caused by these mutations can be observed during embryogenesis, fetal development, at birth, and post-natally [9,10].

The cranial sutures are major growth sites [11] whose fusion occurs either upon completion of early, intense cranial growth or later in life. When sutures close prematurely, specific cranial dysmorphology ensues due in part to the incapacity for growth perpendicular to the closed suture. Mutations that cause premature suture closure occur in genes that are widely expressed throughout development (e.g., FGFRs). These mutations can affect growth and development of diverse tissues [9,10] as the changes in cell signaling initiated by FGFR mutations are not specific to suture mesenchyme and are unrelenting [12], continuing to affect cellular processes and tissue-tissue interactions from conception onwards. Research focused on molecular pathophysiology of premature suture closure has provided much needed information about changes occurring at the subcellular and cellular level local to the suture mesenchyme, but has yet to sufficiently elucidate how these changes translate into the production of more broadly based structural and functional defects.

In recent years, the study of growth and development of skeletal tissue has centered on the study of the genetic control of the temporal sequence of changing osteoprogenitor cell function (e.g., migration, proliferation, differentiation, apoptosis) providing a baseline understanding of the genetic control of these processes (e.g., [2,13]). However morphogenesis of skeletal structure occurs via interactions between cells in multiple tissue layers, including physical forces produced by skeletal and soft tissues that can trigger gene expression to regulate gene function and cell fate. We have previously used precise 3D measures of bone volume, relative bone mineral density, and skull morphology as a guide towards identification of the cellular mechanisms responsible for localized differences between mice carrying particular FGFR2 mutations and their unaffected littermates thereby linking particular aspects of dysmorphogenesis to specific cellular behaviors [14,15]. We have also shown that mutations thought primarily to affect cells of one type (e.g., osteoprogenitor) can have equally profound effects on cells that make up other tissues (e.g., brain, vitreous, skin) [10,16-18], thereby contributing to tissue-level behaviors of morphological development. Advancing our knowledge of the physical aspects of development requires specification of the direction and magnitude of changes required to take an initial morphology to a target shape. Once these architectural details are known, they can become part of a larger model of computational tissue biomechanics that more closely specifies the complement of inputs that contribute to the genetic regulation of development and growth. When quantitatively defined in 3D, growth patterns can be integrated into models used to predict mutation-specific craniofacial phenotypes, to test the veracity of therapeutic agents, or to plan surgical correction.

Prenatal cranial growth patterns are not typically assessed in humans and because nearly all craniosynostosis patients undergo reconstructive surgery during infancy, mouse models offer a valuable resource for addressing the contribution of growth pattern to craniofacial dysmorphogenesis. The premature closure of cranial vault sutures is thought to contribute to late prenatal and postnatal dysmorphogenesis, but the precise interaction of the mutation-driven changes in cellular processes, premature closure of sutures, and growth pattern is not well understood. Here we provide a comparative analysis of craniofacial suture closure and growth pattern in two mouse models for Apert syndrome, each carrying a mutation in FGFR2 that together account for 99% of all known cases of Apert syndrome. We test the hypothesis that cranial growth patterns contribute to cranial dysmorphology in craniosynostosis and demonstrate that differences in a single mutation change in neighboring amino acids cause subtle differences in patterns of skull growth. We quantitatively estimate late prenatal cranial growth patterns and provide a timeline of cranial suture patency patterns using high resolution micro computed tomography (HRμCT) images of the heads of *Fgfr2*^*+/S252W*^ and *Fgfr2*^*+/P253R*^ Apert syndrome mice and their unaffected littermates between embryonic day 17.5 (E17.5) and the day of birth (postnatal day 0, or P0).

## Results

### Skull morphology

**Figure 1:**
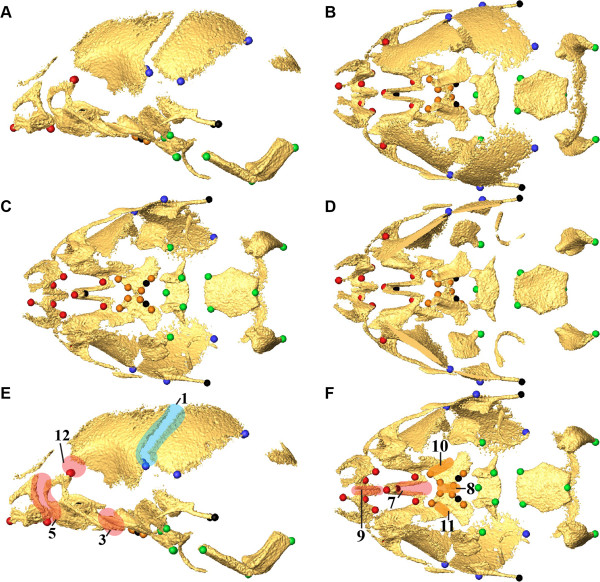
**Landmark and suture placement.** Placement of anatomical landmarks **(A-D)** and sutures **(E,F)** on E17.5 mouse skull. Views are left lateral **(A)**, superior **(B)**, inferior **(C)**, endocranial **(D)**, left lateral **(E)**, and inferior **(F)**. Landmarks **(A-D)** are color coded by region: Face (red); Base (green); Vault (blue); Palate (orange). Additional landmarks that were used only in analysis of the global skull are shown in black. Sutures **(E, F)** are color-coded by region [Face (red); Vault (blue); Palate (orange)] and indicated by number: 1,2) Left, right coronal ; 3, 4) Left, right zygomatic-maxillary; 5,6) Left, right premaxillary-maxillary; 7) Intermaxillary; 8) Interpalatine; 9) Inter Premaxillary; 10, 11) Left, right maxillary-palatine; 12, 13) Left, right fronto-maxillary. Sutures 1-6 and 12-13 are bilateral; only left sutures are shown on the left lateral view **(E)**. An additional table provides more complete definitions of the landmarks, as well as identification of the skull region in which each landmark is located [see Additional file 1]. More information on landmark identification and location can be found at: http://getahead.psu.edu/landmarks_new.html.

**Figure 2:**
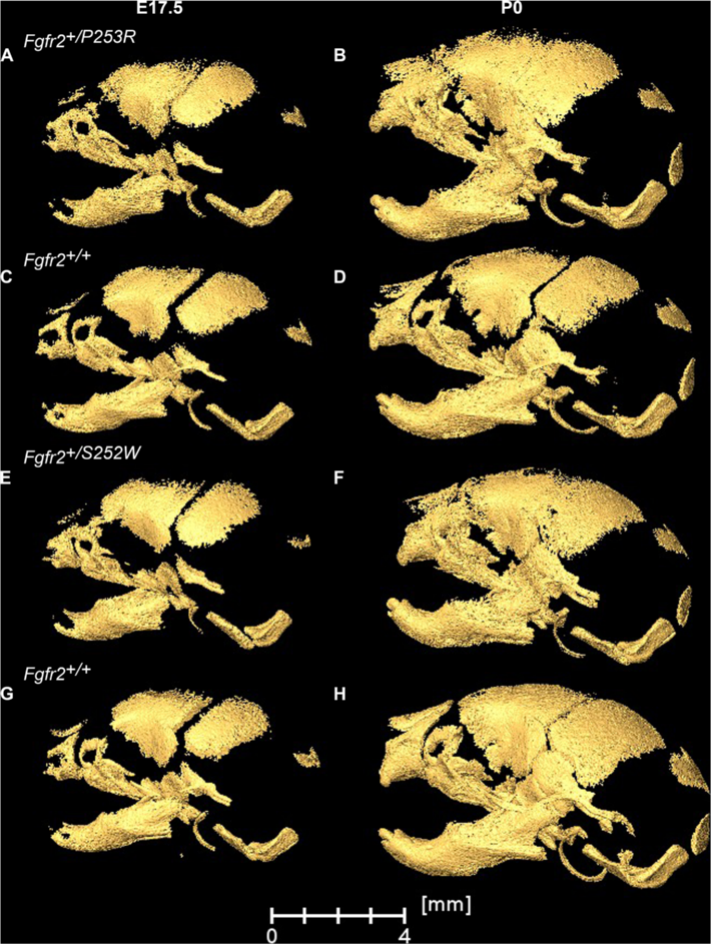
**Skull morphology of Fgfr2**^**+/P253R **^**and Fgfr2**^**+/S252W **^**Apert syndrome mice and unaffected littermates at E17.5 and P0.** Left lateral views of 3D HRμCT reconstructions of representative mice from our study samples: Fgfr2^+/P253R^ mutant at E17.5 **(A)** and P0 **(B)**: unaffected littermate of the P253R model at E17.5 **(C)** and P0 **(D)**; Fgfr2^+/S252W^ mutant at E17.5 **(E)** and P0 **(F)**; unaffected littermate of the S252W model at E17.5 **(G)** and P0 **(H)**. By P0, midfacial retrusion is more severe and fusion of multiple sutures is apparent.

Skull morphology was analyzed at E17.5 and P0 using anatomical subsets of 3D coordinates of skull landmarks representing the entire or global skull, the cranial base, the cranial vault, the facial skeleton, and the palate (Figure 1; An additional table provides complete information for these landmarks [see Additional file 1]). All mice were inbred on C57BL/6 J background to reduce genetic heterogeneity, allowing direct comparison of the impact of each specific FGFR2 mutation (see Materials and Methods). Sample sizes are given in Table 1. Figure 2 provides HRμCT reconstructions of representative individuals from each sample.

**Table 1:** **Samples sizes of *Fgfr2***^***+/S252W ***^**and *Fgfr2***^***+/P253R ***^**Apert mouse models and unaffected littermates (listed to the left of each model) at embryonic day 17.5 (E17.5) and day of birth (P0)**

**Age**							
**E17.5**				**P0**			
***Fgfr2***^***+/+***^	***Fgfr2***^***+/S252W***^	***Fgfr2***^***+/+***^	***Fgfr2***^***+/P253R***^	***Fgfr2***^***+/+***^	***Fgfr2***^***+/S252W***^	***Fgfr2***^***+/+***^	***Fgfr2***^***+/P253R***^
**11**	**10**	**12**	**12**	**25**	**23**	**28**	**36**

**Figure 3:**
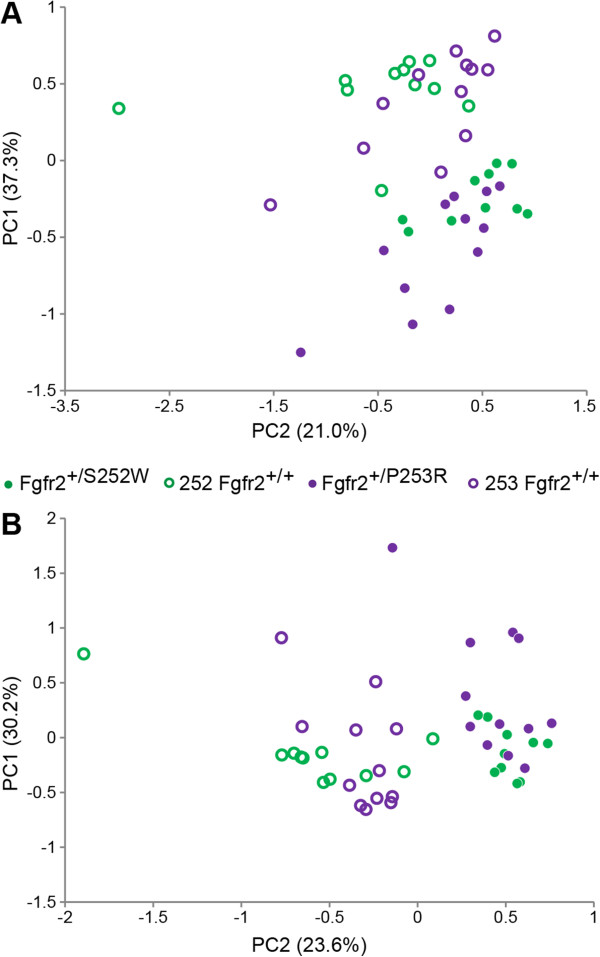
**Principal Components Analysis of form and shape at E17.5. A)** Placement of all E17.5 mouse crania on PC1 and PC2 in the skull form space (as estimated by principal component analysis of all possible linear distances among cranial 33 landmarks on all E17.5 mice). **B)** Placement of all E17.5 mouse crania on PC1 and PC2 in the skull shape space (as estimated by principal component analysis of all possible linear distances of each observation, scaled by the observation’s geometric mean).

**E17.5.** A principal components analysis (PCA) of form based on all 528 unique inter-landmark distances estimated from 33 global skull landmarks (Figure 1), was used as an exploratory first step in describing and comparing cranial morphologies and growth trajectories [19,20]. A plot of the first two principal components of E17.5 mice reveals that cranial morphologies of *Fgfr2*^*+/S252W*^*and Fgfr2*^*+/P253R*^ mice overlap along the first principal component axis (PC1) (accounting for 37% of the total variation in form) and the second axis (PC2) (accounting for 21% of the variation) (Figure 3A). The distribution of mutant cranial forms indicates similarity in the generalized effects of the two mutations on skull shape at E17.5, although *Fgfr2*^*+/S252W*^ cranial morphologies gravitate farther towards the positive ends of PC1 and PC2. The distribution of the *Fgfr2*^*+/P253R*^ mice along PC1 and PC2 suggests increased variation for this mutation group at this age. A clear separation between *Fgfr2*^*+/S252W*^*and Fgfr2*^*+/P253R*^ Apert syndrome mice and their unaffected littermates is seen along PC2. Cranial morphologies of both sets of unaffected littermates show overlap along PC1 and PC2.

To consider the relative amount of variation attributable to shape alone (i.e., without variation due to size) among the E17.5 animals, the PCA was repeated using the linear distances of each observation scaled by the observation’s geometric mean [19,21,22] (Figure 3B). The PCA of the scale-free shape data maintained the separation between mutant and unaffected mice, but only slightly reduced the amount of within-group variation, suggesting that at E17.5, differences in scale do not greatly contribute to form differences between Fgfr2 mutant and unaffected littermates or to the differences between *Fgfr2*^*+/S252W*^*and Fgfr2*^*+/P253R*^ mice.

The 3D patterns of shape difference between the *Fgfr2*^*+/S252W*^ and *Fgfr2*^*+/P253R*^ Apert syndrome mouse models and their unaffected littermates at E17.5 were evaluated using Euclidean Distance Matrix Analysis (EDMA) [23-25] (see Methods). The null hypothesis test of similarity in shape between *Fgfr2* mutant mice and unaffected littermates provide an initial evaluation of form differences between mutant mice and unaffected littermates. An additional table file provides the results of these hypothesis tests [see Additional file 2]. Estimates of localized differences and evaluation of the statistical uncertainty of these estimates are contained within bootstrapped confidence intervals discussed below.

**Figure 4:**
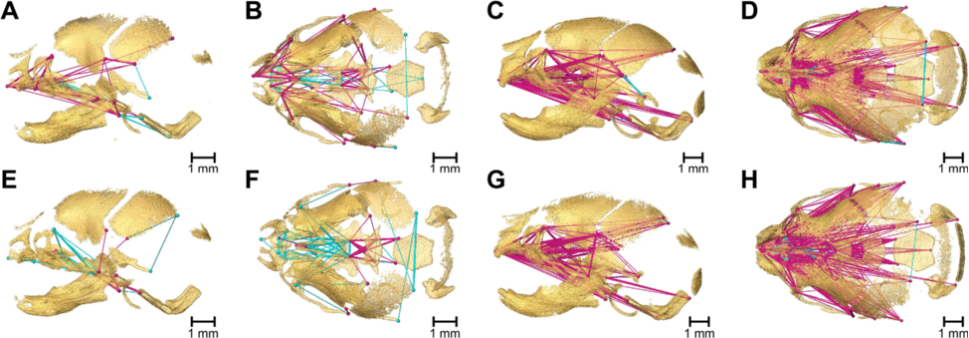
**Morphological variation at E17.5.** Differences in morphology between *Fgfr2*^*+/S252W*^ mice and unaffected littermates at E17.5 (**A**, lateral view; **B**, superior view) and P0 (**C**, lateral view; **D**, superior view) and between *Fgfr2*^*+/P253R*^ mice and unaffected littermates at E17.5 **(E, F)** and P0 **(G, H)**. For all views rostral is left, caudal is right. The linear distances pictured are limited to those that differed significantly by ≥ 5% between mice carrying one of the two Fgfr2 mutations and their respective unaffected littermates (using α = 0.10 confidence limits). The magnitude of these differences varies across the skull. Lines represent distances among landmarks that are significantly larger (blue) and significantly smaller (fuchsia) in mutant mice. Thin lines indicate linear distances that are increased/decreased by 5-10% in mice carrying one of the two Fgfr2 mutations while thick lines indicate linear distances that differ by >10% between unaffected and mutant mice. Bone segmented from HRμCT images is shown as partially transparent to better visualize the differences. See supplementary videos for a full 360-degree rotation view of differences in cranial morphology between *Fgfr2*^*+/S252W*^ mice and unaffected littermates at E17.5 [Additional file 3] and P0 [Additional file 5], and differences in cranial morphology between *Fgfr2*^*+/P253R*^ and unaffected littermates at E17.5 [Additional file 4] and P0 [Additional file 6].

Confidence intervals for local effects of the two *Fgfr2* Apert syndrome mutations at E17.5 reveal differential characteristics of mutation-driven shape change. Most notable is the overall reduction in the more rostral elements of the skull in *Fgfr2*^*+/S252W*^ mice relative to unaffected littermates (Figure 4A, B), especially in the premaxillae, the posterior aspect of the palate, and dimensions that connect the premaxillae with the rostral cranial base. An additional movie file provides a 3D view of these differences [see Additional file 3]. In contrast, *Fgfr2*^*+/P253R*^ mice show localized increases in posterior facial dimensions relative to unaffected littermates (Figure 4E, F). An additional movie file provides a 3D view of these differences [see Additional file 4]. Both models show a rostrocaudal reduction across the basioccipital synchondrosis, coupled with increasing distances between the rostral aspect of the basioccipital and the pterygoid processes. *Fgfr2*^*+/P253R*^ mice show an increase in distances between the frontal process of the maxilla and the palate relative to unaffected littermates (Figure 4E, F), which is unique to this mutation. A relative increase in width of the caudal cranial vault is seen in both models (Figure 4B, F). *Fgfr2*^*+/P253R*^ mice also show an increase in caudal cranial vault height (Figure 4E).

**P0.** At P0, *Fgfr2*^*+/S252W*^ (Figure 4C, D) and *Fgfr2*^*+/P253R*^ (Figure 4G, H) mutant mice demonstrate statistically significant differences in global shape and in the shapes of all anatomical subsets relative to their respective littermates [Additional file 2]. A four to five-fold increase in the number of significant differences of local cranial measures are present at P0 relative to what is estimated for morphological differences at E17.5. Additional movie files provide a 3D view of these differences [see Additional files 5 and 6]. Details of the specific differences in the anatomical effects of the two Apert syndrome mutations at P0 were previously described [14,26-28] and differ from those described for E17.5. At P0, differences between mutant mice and their unaffected littermates occur across the entire skull but the facial skeleton is the most affected region, with *Fgfr2*^*+/S252W*^ mutant mice displaying significantly more severe dysmorphology localized to the posterior palate [14,26]. Coronal suture patency was not strongly correlated with skull dysmorphology at P0 in either Fgfr2 mutant.

### Growth related shape variation

**Figure 5:**
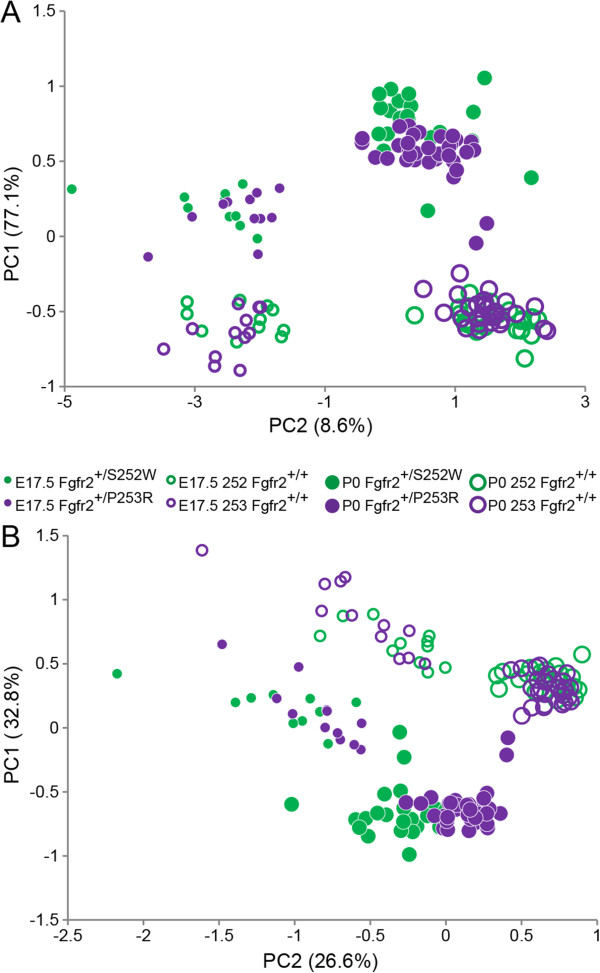
**Principal Components Analysis of form and shape: E17.5 and P0 data combined. A)** Placement of E17.5 and P0 mouse crania on PC1 and PC2 in the skull form space as estimated by principal component analysis of all linear distances among 33 cranial landmarks. **B)** Placement of the all E17.5 and P0 mouse crania on PC1 and PC2 in the skull shape space (as estimated by principal component analysis of all possible linear distances scaled by the age and genotype-specific geometric sample mean) among cranial 33 landmarks on all E17.5 and P0 mice.

Principal components analyses of ontogenetic variation in skull form (see Methods) revealed that skull morphology of mice carrying the Fgfr2 S252W and P253R mutations cluster near one another at both E17.5 and P0, clearly separated from their age-matched unaffected littermates (Figure 5A). PC1 (accounting for 77% of variation in form) primarily reveals the impact of developmental age, with the skulls of all E17.5 mice gravitating towards the negative end of PC1, and the older mice situated at the positive end (Figure 5A). PC2 (accounting for ~8% of the variation in craniofacial form) separates groups according to whether or not group members carry one of the two Fgfr2 mutations. Although the morphological consequences of developmental age convey a strong signal during late embryogenesis (characterized by PC1), the impact of carrying one of the two Fgfr2 mutations is also apparent at E17.5 and at P0 (characterized by PC2).

An alternate way to consider the relative amount of variation in shape among the ontogenetic samples is to repeat the PCA analysis using linear distances for each observation scaled by their respective geometric mean (see Methods). Separation of groups is maintained in the analysis of skull shape, though the distance among groups is decreased, emphasizing both the substantial contribution of size-related changes in shape to variation produced by growth and the distinct patterns of dysmorphogenesis associated with these *Fgfr2* mutations (Figure 5B). Distinction among groups defined on the basis of developmental age is delineated primarily along PC1 (accounting for ~33% of variation in shape), but an equally clear separation among skull shapes defined by the presence/absence of an Fgfr2 mutation is revealed along PC2 (~27% of variation in shape), indicating that a substantial proportion of variation in skull shape is a consequence of carrying one of the two Fgfr2 mutations. Approximately one-third of the overall variation is due to shape alone, as calculated by the ratio of the sum-of-variances for *ln*-transformed ratios of distances to their geometric mean (1.18) to the sum-of-variances for *ln*-transformed distances among all landmarks (3.75) - a remarkable amount given that data from two age groups comprise substantial differences in overall size [19,21]. Regardless of whether cranial form (Figure 5A) or cranial shape (Figure 5B) is considered, distinction between the *Fgfr2*^*+/P253R*^ and *Fgfr2*^*+/S252W*^ Apert syndrome mice cranial morphologies is not apparent at E17.5, but the two mutation groups are fairly well-discriminated at P0.

### Growth difference of Apert syndrome *Fgfr2*^*+/S252W*^ and *Fgfr2*^*+/P253R*^ mice

**Figure 6:**
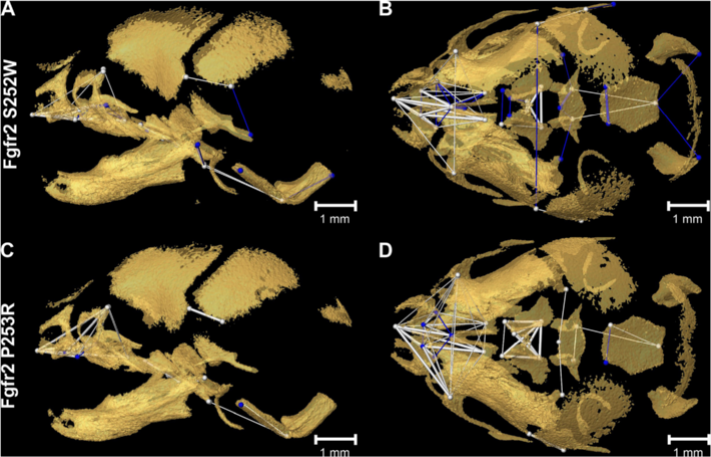
**Variation in growth of Apert syndrome models.** Differences in growth between *Fgfr2*^*+/S252W*^ and unaffected littermates (**A**, lateral view; **B**, superior view) and *Fgfr2*^*+/P253R*^ and unaffected littermates (**C**, lateral; **D**, superior). For all views rostral is left, caudal is right. The linear distances pictured are limited to those whose growth from E17.5 to P0 differed significantly by ≥ 5% using α = 0.10 confidence limits. The magnitude of these differences in growth varies across the skull. Linear distances that grew significantly more (blue) and significantly less (white) in mutant mice are shown. Thin lines indicate linear distances whose growth was increased or decreased by 5-10% in mice carrying one of the two Fgfr2 mutations relative to their respective unaffected littermates; thick lines indicate linear distances whose growth differed by >10% between unaffected and mutant mice. Bone segmented from HRμCT images is shown as partially transparent to better visualize the growth differences. See supplementary videos for a full 360-degree rotation of differences in cranial growth between *Fgfr2*^*+/S252W*^ mice and unaffected littermates [Additional file 8] and between *Fgfr2*^*+/P253R*^ and unaffected littermates [Additional file 9].

We quantify growth as the process that changes the configuration (size and shape) of a mouse skull at E17.5 to its configuration at P0 and statistically evaluate differences in growth among groups using Growth Difference Matrix Analysis (GDMA) (see Methods). Within each model for Apert syndrome, mice carrying an *Fgfr2* mutation exhibited statistically significant differences in late prenatal skull growth relative to unaffected littermates for most of the craniofacial regions. An additional table provides the results of these hypothesis tests [see Additional file 7]. Confidence intervals testing reveal statistically significant differences in growth of the majority of linear distances measured among landmarks. Relative to unaffected littermates, *Fgfr2*^*+/S252W*^ and *Fgfr2*^*+/P253R*^ Apert syndrome mice both display decreased magnitudes of growth in most rostrocaudal dimensions crossing the premaxillae and the maxillary palatal shelves (Figure 6). Additional movie files show these growth differences in 3D [see Additional file 8 for 3D view of differences in growth between *Fgfr2*^*+/S252W*^ mice and unaffected littermates and Additional file 9 for 3D view of differences in growth between *Fgfr2*^*+/P253R*^ mice and unaffected littermates]. Growth is also diminished along distances between the most dorsal extension of the frontal process of the maxillae and points on the rostral cranial base and palate in both models contributing to a facial skeleton in mutant mice that is generally narrowed, flattened and reduced in height. Relative to unaffected littermates, growth is increased in distances between the ethmoid and the premaxillary-maxillary suture (Figure 6B, D). The relative increase in mediolateral growth of the cranial base and neurocranium seen in *Fgfr2*^*+/S252W*^ mice (Figure 6A,B; Additional file 8) does not occur in *Fgfr2*^*+/P253R*^ mice (Figure 6C,D; Additional file 9). Relative to unaffected littermates, both models experience increased growth of the caudal cranial base along a rostrocaudal axis.

The most caudal aspect of the palate has been previously identified as a site of localized dysmorphology in *Fgfr2*^*+/S252W*^ and *Fgfr2*^*+/P253R*^ Apert syndrome mice, as well as a region where the morphological effects and localized cellular processes of the two Fgfr2 mutations can be differentiated [14]. Hypothesis tests of similarity in growth show that palatal growth patterns are different from unaffected mice in both Apert syndrome models. Direct comparison of palatal growth between *Fgfr2*^*+/S252W*^ and *Fgfr2*^*+/P253R*^ Apert syndrome mice revealed high variability in palatal growth for each model. As a result, even though the magnitude of growth of some palatal dimensions were dissimilar between the two models, the differences were not significant (Additional file 2). Compared to unaffected littermates, differences in growth are strongest at the caudal aspect of the pterygoid plates in both models.

Relative to unaffected littermates, magnitudes of late prenatal growth of the cranial base in *Fgfr2*^*+/S252W*^ Apert syndrome mutant mice are significantly increased along mediolateral dimensions. Only the increased mediolateral growth of the basi-sphenoid synchondrosis is shared with *Fgfr2*^*+/P253R*^ Apert syndrome mice; the other local changes in cranial base growth are specific to the FGFR2 S252W mutation (Figure 6). Differences in growth of the cranial vault are minimal between *Fgfr2*^*+/S252W*^*and Fgfr2*^*+/P253R*^ Apert syndrome mice and their unaffected littermates (Figure 6).

### Estimation and evaluation of hypothetical forms

Differences in cranial morphology among our samples at P0 are due to the combination of altered morphology at E17.5 and distinct growth patterns for each genotype group. The production of hypothetical forms and their evaluation with respect to samples of known morphologies [24,29] provide an alternate approach to the evaluation of the contribution of growth pattern to craniofacial phenotypes. The hypothetical forms in this case embody the 3D morphology of an unaffected mouse cranium at E17.5 and the growth pattern estimated for mice carrying one of the two *Fgfr2* mutations. The 3D coordinates of the two hypothetical crania (A: the average cranial morphology of an unaffected littermate at E17.5 grown using the *Fgfr2*^*+/S252W*^ mutant growth pattern; and B: the average cranial morphology of an unaffected littermate at E17.5 grown using the *Fgfr2*^*+/P253R*^ mutant growth pattern) were added to our samples and analyzed using PCA. Because the simulated morphologies (Hypothetical forms A and B) and the unaffected mice share the same initial morphologies at E17.5*,* any differences observed between the hypothetical forms and unaffected P0 mice can be attributed exclusively to differences in growth caused by the presence of an *Fgfr2* mutation.

**Figure 7:**
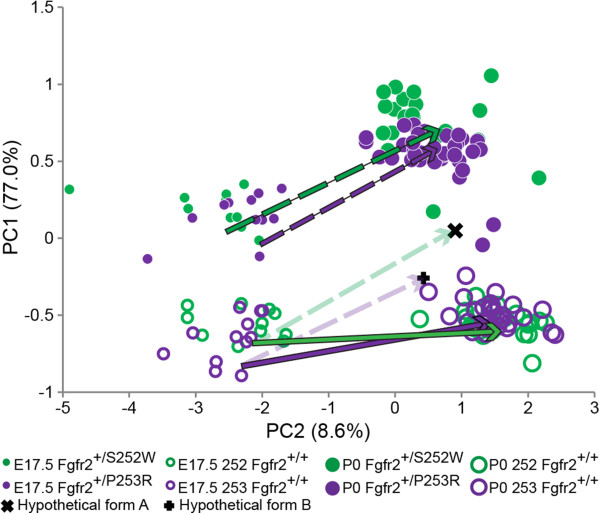
**Mouse cranial in the skull form space.** Placement of all mouse crania on PC1 and PC2 in the skull form space estimated by PCA of all unique linear distances among 33 cranial landmarks (on all mice including two hypothetical forms) to show the estimated growth trajectory for each sample as vectors. GDMA revealed growth patterns between mutant mice and unaffected littermates to be statistically different (Figure 6; Additional file 7). Group-specific mean vectors begin at the location of the E17.5 group mean form in the skull form space defined by the PCA and end at the location of the P0 group-specific mean form. Hypothetical form A represents the average *Fgfr2*^*+/+*^ (S252W) cranial morphology grown using the *Fgfr2*^*+/S252W*^ growth pattern. Hypothetical form B represents the average *Fgfr2*^*+/+*^ (P253R) cranial morphology grown using the *Fgfr2*^*+/P253R*^ growth pattern. The growth trajectories for the hypothetical forms (ghosted) begin at the group-specific unaffected E17.5 mean and are equivalent in direction and magnitude to the growth trajectories of the mutant mice, ending at the position of the hypothetical forms (see text).

PCA is used to visualize differences in growth trajectories (Figure 7) previously shown by GDMA to be statistically different between mutant and unaffected groups. Neither hypothetical form falls within the concentration of unaffected P0 cranial morphologies, revealing the contribution of abnormal prenatal growth pattern to the morphological differences between genotypes at birth. The position of the two hypothetical forms suggests that the *Fgfr2*^*+/S252W*^ mutation provides a relatively stronger contribution to dysmorphogenesis.

### Patterns of suture patency

**Figure 8:**
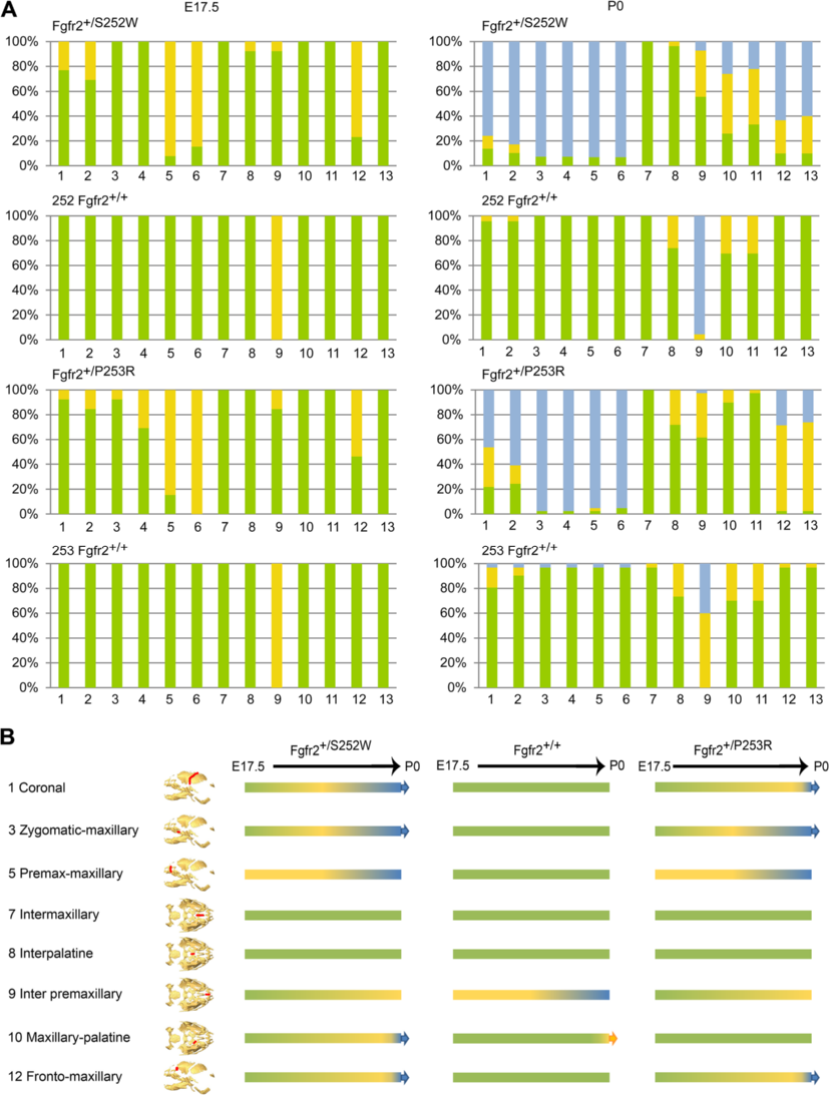
**Craniofacial suture patency in *Fgfr2***^***+/S252W***^**and *Fgfr2***^***+/P253R***^**mice and unaffected littermates visualized by HRμCT. A)** Patent sutures are green, partially patent (closing) sutures are yellow, and sutures that are no longer patent (closed) are blue. Suture identification is along the X-axis while the percent of individuals within a sample showing a patency state is given on the Y-axis. Numeric codes for the sutures: 1, 2 – Left, right coronal; 3, 4 – Left, right zygomatic-maxillary; 5,6 – Left, right premaxillary-maxillary; 7 - Intermaxillary; 8 - Interpalatine; 9 - Inter premaxillary; 10, 11 – Left, right maxillary-palatine; 12,13 – Left, right fronto-maxillary. Suture locations are shown in Figures 1 and 8B. Only coronal suture patency was recorded for the neurocranium as all other neurocranial sutures were patent in all animals at these ages. The internasal suture was patent in all mice. **B)** Timeline of cranial suture closure based on data from E17.5 and P0 mice. Colors used on trajectories between the observed time points are interpolated. Arrows at the end of trajectories indicate that the status observed at P0 is ongoing. Trajectories without an arrow suggest that future suture patency states could not be interpolated from available data. Suture patency patterns were similar across unaffected littermates so data were pooled.

Current theories state that when premature fusion of cranial vault sutures occur, secondary distortion of skull shape results in part from the lack of growth perpendicular to the fused suture and compensatory overgrowth at the non-fused sutures [30]. To test whether these same principles apply to sutures of the facial skeleton we scored patterns of suture patency as visualized on HRμCT images of each mouse assigning qualitative scores of open, partially open, or fused to the entire length of the sutures (Figure 8A). These observations were used to build a comparative timeline of suture closure in all groups of mice (Figure 8B).

We previously demonstrated a higher percentage of *Fgfr2*^*+/S252W*^ mice with complete (69%) bicoronal synostosis at P0 compared to *Fgfr2*^*+/P253R*^ mice [26]. At E17.5, variable levels of coronal suture fusion are seen in both Apert syndrome models (Figure 8A, columns 1,2) although neither displays completely fused coronal sutures, indicating that premature fusion of coronal sutures is well underway at E17.5 and are nearing complete fusion by P0 [26]. The zygomatic-maxillary and premaxilla-maxillary sutures are invariably fused in *Fgfr2*^*+/S252W*^*and Fgfr2*^*+/P253R*^ mutant mice at P0, while these sutures are consistently patent (at least partially) in unaffected P0 littermates (Figure 8A, columns 3,4,5,6). No fusion of the zygomatic-maxillary sutures is seen in *Fgfr2*^*+/S252W*^ mutant mice at E17.5, but unilateral and bilateral partial fusion is seen in small numbers of *Fgfr2*^*+/P253R*^ mutant mice at E17.5 (Figure 8A, columns 3,4). A majority (85%) of *Fgfr2*^*+/P253R*^ and *Fgfr2*^*+/S252W*^ mutant mice show bilateral partial fusion of the premaxilla-maxillary suture at E17.5 while these sutures are fully patent in unaffected littermates (Figure 8A, columns 5,6). The maxillary-palatine sutures are patent in all animals at E17.5 but show variable patterns of fusion at P0 (Figure 8A, columns 10,11). The fronto-maxillary sutures show a consistent tendency towards fusion in *Fgfr2*^*+/S252W*^*and Fgfr2*^*+/P253R*^ mice at P0 (Figure 8A, columns 12,13), but remain completely patent in unaffected littermates. Most midline palatal sutures (Figure 8A, columns 7,8,9) are patent in all mice at E17.5, but the interpremaxillary suture reveals a tendency towards fusion in unaffected mice beginning at E17.5 and continuing to P0 (Figure 8A, column 9).

**Figure 9:**
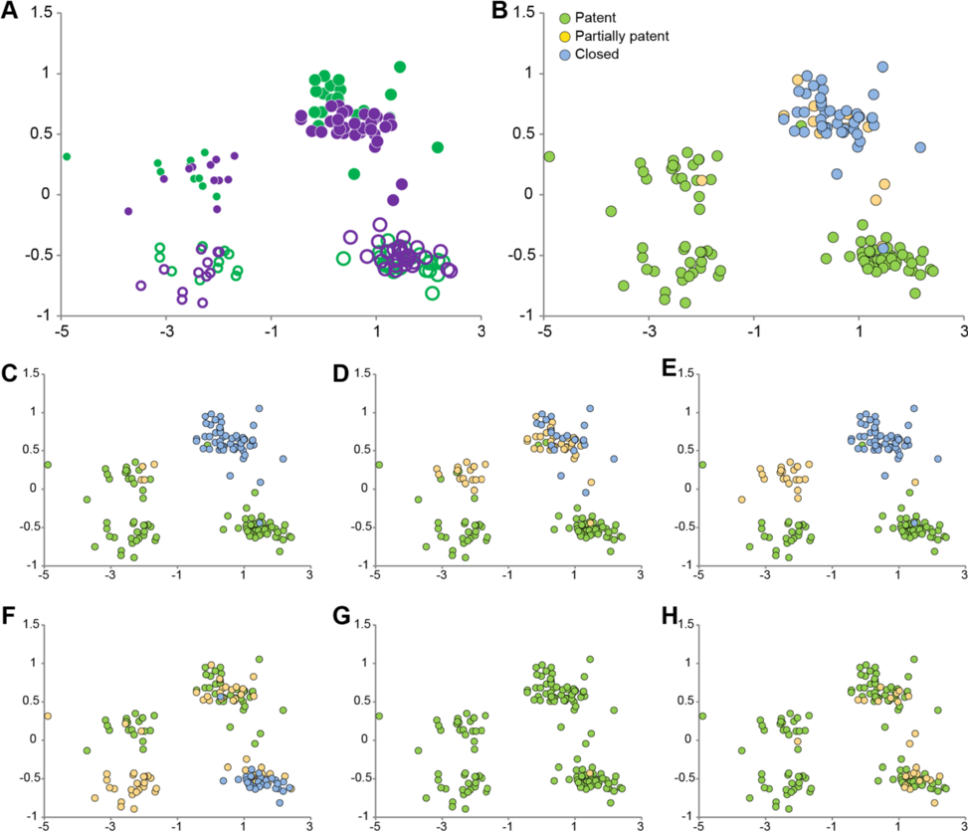
**Relationship of suture patency patterns and craniofacial shape as estimated by PCA. (A)** Distribution of all individuals along PC1 and PC2 following Figure 5A. (see Figure 5A and Methods for details of computing the PCA). **(B)** Distribution of individuals along PC1 and PC2 coded by the average patency of all facial sutures (coronal suture patency is not included in this average); **(C-H)** Distribution of individuals along PC1 and PC2 coded for patency of: zygomatic-maxillary suture **(C)**; frontomaxillary suture **(D)**; premaxillary-maxillary suture **(E)**; inter-premaxillary suture **(F)**; inter-maxillary suture **(G)**; inter-palatine suture **(H)**.

To explore the possibility that compensatory growth driven by the premature closure of facial sutures drives craniofacial dysmorphology, we examined the relationship between facial suture closure patterns and growth related cranial shape variation. States of facial suture patency (open, partially closed, closed) coded for individual mice were plotted on the basis of differences in skull shape as quantified by PCA (Figure 9). The average condition of all facial sutures estimated for each mouse reveals a strong relationship between facial suture patency and intensity of craniofacial dysmorphology (Figure 9B) while the relationship between patency of individual facial sutures and cranial shape variation (Figures 9C-H) reveals local patterns. Closing or closed bilateral facial sutures are always associated with increased facial dysmorphology as defined by PC2 (Figure 9C-E), while the relationship between midline sutures (that show varying patterns of patency) and facial dysmorphology is less clear. The abnormally patent interpremaxillary suture in mutant mice (Figure 9F) enables compensatory mediolateral facial growth not possible in unaffected mice.

## Discussion

Although the specific changes in craniofacial shape and the magnitude and direction of 3D cranial growth patterns in mice do not coincide exactly with those of human beings, our results agree with observations of infant Apert syndrome phenotypes. In humans, Apert syndrome cases carrying the *FGFR2* S252W mutation have a more severe facial phenotype relative to those who carry the *FGFR2* P253R mutation, while the *FGFR2* P253R group has more severe limb anomalies [5,31,32]. These craniofacial findings have recently been confirmed quantitatively in a study of craniofacial phenotypes in FGFR-related craniosynostosis syndromes [33]. Our analysis concurs with these observations of the differential effects of the two Apert syndrome mutations revealing that mice carrying the Fgfr2S252W mutation typically have a more severe facial phenotype and a growth pattern that contributes to the increased intensity of facial dysmorphology relative to mice carrying the Fgfr2P253R mutation.

Mutation-driven differences in growth patterns concur with the observations that growth modification in Apert syndrome has its onset prenatally [34,35], and that dysmorphology increases in severity with age, producing additional complications [36]. Because of the continual contribution of FGF/FGFR signaling to cell processes that direct the assembly and growth of diverse cranial tissues, FGFR mutations affect growth patterns in unpredictable ways. Individuals born with Apert syndrome invariably face multiple complex surgeries that begin in infancy and continue through adulthood. The functional and cosmetic outcomes of these procedures cannot be anticipated in detail and the strategy and number of these surgeries are typically tailored to the individual. We have provided evidence that variation in the causative mutation, the severity of disease phenotypes, and the changes that occur with growth of the craniofacial complex might be significant contributing factors to the uncertainty of long-term surgical outcome. Individual phenotypes result from change(s) in specific (and potentially diverse) signalling and regulatory cascades, and these changes have consequences for development of various tissues and, ultimately craniofacial variation. Quantitative definition of growth patterns, the ability to anticipate these trajectories, and the use of evidence-based growth simulation in patient care could contribute to improved patient-centered outcomes either through changes in surgical approach, or through more realistic modeling and expectation of surgical outcome. Our results indicate that growth pattern is an additional source for observed phenotypic differences among patients carrying these differing Apert syndrome mutations and suggest that developed therapies need to specifically target the mutation rather than being generally adapted to the disease.

Previous work with mouse models has revealed that tissues other than bone are affected by *Fgfr2* mutations [10,16] and that early postnatal growth patterns of the brain and skull in *Fgfr2*^*+/P253R*^ Apert syndrome mice differ from their unaffected littermates [37]. The impact of growth pattern on emergent prenatal morphology quantified here is suggestive of the combination of two origins of growth insult: 1) mutation-driven disruption of early cellular processes (e.g., cell migration, proliferation, differentiation) resulting in the initial construction of abnormal phenotypes; coupled with 2) the impact of the mutation on additional cellular processes and tissue-tissue interactions resulting in changes in functional pressures and exchanges among growing tissues, changes often considered secondary to the direct mutational effects. Early closure of facial sutures, a direct result of disrupted cellular processes, constitutes yet another influence on growth but due to the comparative abundance of sutures within the facial skeleton, the ‘compensatory growth’ model described in the case of prematurely closed vault sutures [38] is not easily translated to facial growth patterns in craniosynostosis syndromes.

Suture formation, patency and eventual closure occurs through coordinated integration of signaling pathways (e.g., FGF, TGFβ, Wnt) via processes that are not currently understood. Our inability to identify the physiological trigger that elicits premature suture closure indicates that the molecular instructions for this event and for suture closure in typical cases occur well within hierarchies of interactions that involve genes carrying identified mutations. Premature closure of sutures is an example of “tinkering” [39] of the evolutionarily stable, generic suture fusion event, though patency/closure of each suture may be supervised by subtly different change(s) in specific regulatory cascades. Vault and facial suture patency may be sensitive to similar or different signaling families, to variation in the strength or timing of signals, or to shifts in patterning boundaries. Changes in patency patterns can have multiple consequences for phenotypic variation.

Evolutionarily, sutures are recognized as much for their role in mediating cranial mechanics as for their contribution to bone growth [40]. Vertebrate evolution reveals a general trend of reduced cranial kinesis through the loss or restriction of some intracranial joints (sutures) and reduction in the number of jaw and skull bones (and concurrently the number of sutures) [41], which is strongly associated with changes in cranial morphology. Though it is not possible to record changes in rates of suture closure across evolutionary time scales, our systematic record of differences in relative rates of suture closure in mutant and typically developing mice strongly suggests a role for variation in the relative rates of suture closure (and growth at sutures) in generating phenotypic variability in craniofacial shape over evolutionary time.

Evidence presented here of the local and global aspects of 3D growth patterns that contribute to craniofacial morphogenesis provides a layer of information rarely quantified in the dissection of mutational effects on phenotypes. Current research paradigms focused on the mechanistic basis of premature suture closure reveal highly localized effects of activating mutations (e.g., [14,27,28,42-44]), but these effects vary by suture (and even by location within the suture) and time of development. In the case of FGFR-related craniosynostosis syndromes, FGFR mutations affect many tissues throughout development [10]. Local patterns of signaling are dynamic, continually changing in intensity and temporo-spatial distribution such that current methods of histological and immunohistochemical observation can provide only a limited sampling of data required to predict morphology. Because of the continual contribution of FGF/FGFR signaling to cell processes that direct the assembly and growth of diverse cranial tissues, FGFR mutations affect growth patterns in ways that are currently unpredictable. Groups of cells, tissues and organs respond to the mutant signal by changing their behavior according to the aberrant instruction, but must simultaneously adjust behaviors in response to the signals emanating from functional pressures of surrounding cells and tissues, including the shifting constraints caused by prematurely closing sutures. Complex combinations of molecular, cellular and biomechanical information underlying patterns of accommodation of developing structures constitute the growth pattern. Combining precise knowledge of mutation-induced changes in growth pattern with geometric information of local patterns of changes in signaling and cell function using a multiscale computational network approach can contribute to models of developmental tissue mechanics bringing us closer to modeling testable hypotheses of the morphogenetic unfolding of genetic programs during ontogeny.

## Conclusions

Our results demonstrate that the differential effects of the FGFR2 S252W and P253R mutations result in mutation-specific prenatal cranial growth patterns that are responsible, at least in part, for differences in postnatal cranial phenotypes of the two mutation groups. We have demonstrated that the two gain-of-function mutations in FGFR2 uniquely perturb prenatal cranial growth patterns and cause differences in suture patency and craniofacial dysmorphology that can be quantitatively distinguished as early as E17.5. Within each mutation group, the cranial features that are divergent between mutant and unaffected littermates at E17.5 are not the same features that are divergent at P0. Further, the patterns of growth suggest that dysmorphologies become increasingly different with age. Finally, regardless of initial morphology, we demonstrate how precise 3D estimates of changes in the growth pattern caused by the presence of either of these two *Fgfr2* mutations contribute to the production of craniofacial dysmorphology.

Our findings suggest that direct and indirect effects of a mutation need to be considered when determining the production of craniofacial morphology. Individual variation in FGFR signal strength and response-to-signal contribute to osteoprogenitor cell differentiation, bone deposition, and the premature fusion of sutures, but physical accommodations of changing structures over time also participate and contribute to signaling cascades that are part of cranial morphogenesis. Any proposed therapeutic strategy that targets cellular processes local to a site of altered development (e.g., a suture) should be evaluated in terms of the effects of that localized rescue on surrounding tissues, and the impact of local changes in cell dynamics on growth of the overall cranial assembly. Although our results specifically address disease-causing FGFR2 mutations demonstrating the relevance of altered growth pattern to the production of FGFR-associated dysmorphogenesis, this approach can be applied to other murine disease models and to human data when available.

## Methods

### Generation of targeting construct and Apert FGFR2 mouse models

Our sample consists of *Fgfr2*^*+/S252W*^ and *Fgfr2*^*+/P253R*^ Apert syndrome mouse models and their unaffected littermates (Table 1), generated at Johns Hopkins Medical Institutions and Mount Sinai Medical Center [27,28]. Both knock-in mouse models have been back-crossed onto the same genetic C57BL/6J background for more than ten generations, allowing direct comparison between models. Embryos harvested at E17.5 and P0 mice were euthanized by inhalation anesthetics and fixed in 4% paraformaldehyde. Gestation time was 19.0 ± 0.5 days. Genotyping of tail DNA by PCR was performed to distinguish mutant from unaffected littermates [27,28]. Mouse litters were produced, sacrificed and processed in compliance with animal welfare guidelines approved by the Johns Hopkins University, the Mount Sinai Medical Center and the Pennsylvania State University Animal Care and Use Committees.

### Image acquisition and landmark data collection protocols

Our sample contained 112 P0 mice and 45 E17.5 embryos (Table 1). High-resolution micro-computed tomography (HRμCT) images with pixel size and slice thickness ranging from 0.014 to 0.025 mm were acquired by the Center for Quantitative X-Ray Imaging at the Pennsylvania State University (http://www.cqi.psu.edu) using the HD-600 OMNI-X high-resolution X-ray computed tomography system (Bio-Imaging Research Inc, Lincolnshire, IL). Image data were reconstructed on a 1024 x 1024 pixel grid as a 16-bit TIFF but were reduced to 8-bit for image analysis. Isosurfaces were reconstructed to represent all cranial bone at P0 or E17.5 based on hydroxyapatite phantoms imaged with the specimens using the software package Avizo 6.3 (Visualization Sciences Group, VSG). The minimum thresholds used to create the isosurfaces ranged from 70-100 mg/cm^3^ partial density hydroxyapatite. A set of 33 three-dimensional (3D) landmarks describing the global skull (Figure 1; Additional file 1) was collected from the isosurfaces. Each specimen was digitized twice by the same observer and measurement error was minimized by averaging the coordinates of the two trials. The maximum accepted error in landmark placement was 0.05 *mm*.

### Statistical evaluation of shape and growth differences

#### Comparison of morphologies

**Principal components analysis of form and shape.** Ontogenetic variation in skull shape was initially assessed using principal components analysis (PCA), an approach that summarizes the variation of a large number of variables (528 unique interlandmark distances estimated from 33 landmarks representing the global skull for each individual) in a lower-dimensional space. The low-dimensional space is defined by principal components axes, which are mutually-orthogonal, linear combinations of the measurement data. The scores of an observation along the principal axes map that observation into the space. When ontogenetic samples are considered, growth trajectories can be visualized and compared by plotting the scores along two or more principal axes.

Two types of PCA were carried out: a PCA based on variation in form (size and shape together), followed by a PCA based on shape variation alone [19,21,22]. For form, all of the inter-landmark distances were *ln*-transformed and their variance-covariance matrix was used as the basis for the PCA. For shape alone, the linear measures were used to define dimensionless shape variables, where all information about the absolute size of the measurements was removed and only information about proportions remained. The shape variables for an observation were defined as the *ln*-transformed ratios of its linear distances to the geometric mean of all of its distances (where the geometric mean serves as a measure of overall size). As with the PCA for form, the PCA for shape was based on the variance-covariance matrix. When using these definitions, the amount of overall variance in form can be partitioned into the proportion that is due to form (size and shape) variation and the proportion that is due to variation in shape alone [19]. The amount of variation due to form (size and shape) is the sum of variances for all of the *ln*-transformed linear measurements, while the amount of variance due to shape alone is the sum of variances for the *ln*-transformed ratios. The difference in these is the amount of variance due to size alone. All principal components analyses were performed using SAS 9.3 (SAS Institute, Cary, NC).

**Confidence intervals for differences in form.** The global skull was defined by the 3D coordinates of the 33 landmarks with subsets of these landmarks used to define the face, cranial vault, cranial base and palate (Figure 1, Additional file 1). We used Euclidean Distance Matrix Analysis (EDMA; [24]) to statistically evaluate shape differences between samples of mutant mice and unaffected littermates as well as to assess differences in growth patterns. EDMA is a 3D morphometric technique that is invariant to the group of transformations consisting of translation, rotation, and reflection [23,29]. Briefly, the original 3D coordinates of landmark locations representing the forms are re-written and analyzed as a matrix of all unique linear distances among landmarks called the form matrix or *FM*. An average *FM* is estimated for each sample following [24]. The form difference between samples is evaluated by calculating ratios of like-linear distances using the average *FM*s of the two samples. The resulting matrix of ratios, the form difference matrix (*FDM*), represents the collection of relative differences among the distances used to define the forms. A non-parametric bootstrap procedure is used to obtain confidence intervals for elements (each corresponding to a linear distance) of the *FDM*[23,24]. Confidence interval testing reveals the localized effects of the mutations on the craniofacial skeleton. We also include a non-parametric statistical test of the null hypothesis of similarity in form between the samples for 3D landmark datasets representing both the entire (global) skull and anatomical sub-regions (face, cranial vault, cranial base, palate)[see Additional file 2]. For each group of landmarks representing regions of the skull, an *FDM* is estimated. The ratio of the maximum entry of the *FDM* to the minimum entry (maximum ratio of inter-landmark distances divided by minimum ratio, or *max/min*) serves as a test statistic measuring the degree of difference between forms. It is evaluated using a non-parametric bootstrap (100,000 resamples) to assess the null hypothesis that the mean form of the two samples is the same [24]. If *max/min* falls in the extreme right-hand tail of the null distribution, we reject the null hypothesis at the appropriate level of significance. We report the p-values in Additional file 2. Note that even when the hypothesis of similarity in form for a cranial region cannot be rejected, confidence intervals may indicate specific linear distances that are statistically different between the samples under investigation revealing highly localized significant differences between samples. All EDMA analyses were performed using EDMAware [45].

#### Statistical comparison of growth patterns

Because size and shape are inextricably linked in growth, we adopt a methodology for the quantitative analysis of growth patterns that moves the analysis of growth to the 3D form space of biological objects using growth difference matrix analysis (GDMA), to statistically compare growth patterns following [24,29]. As described for the comparison of forms (above), 3D landmark locations are used to estimate a *FM* for two samples (here representing different age groups), and ratios of like linear distances are used to estimate the relative changes in geometry that occur due to growth. To estimate the growth matrix, or *GM* for each group, the *FM* of the newborn mouse serves as the numerator and the *FM* of the E17.5 sample serves as the denominator, and ratios are estimated element-wise. In our application, a *GM* is calculated within each genotype group (*Fgfr2*^*+/P253R*^ mice; P253R unaffected littermates; Fgfr2^*+/S252W*^ mice; S252W unaffected littermates) as an estimate of the change due to growth from E17.5 to P0. The *GM* estimates growth for each sample as the relative change in the lengths of all unique linear distances between landmarks. Differences in growth between samples are estimated by the growth difference matrix (*GDM*) consisting of the element-wise ratios of the *GM* estimated for mutant mice to the *GM* estimated for unaffected littermates. Elements of the GDM are statistically evaluated using methods similar to those described above for form using non-parametric bootstrapping (100,000 resamples) and confidence interval testing (α = 0.10) [24,29] (Figure 6). Growth differences were also estimated and statistically evaluated for anatomical regions [see Additional file 7].

#### Hypothetical forms

We used EDMA to produce hypothetical forms from 3D morphological data by applying the *GM* calculated for a sample (e.g., growth of *Fgfr2*^*+/P253R*^ mice from E17.5 to P0) to the *FM* representing the average form from another sample (e.g., FGFR2 *P253R* unaffected littermates at E17.5) following [29]. Hypothetical forms are produced by an element-wise multiplication of the *FM* of interest (the E17.5 starting form) by the *GM* of interest (the growth trajectory), with both matrices written as vectors. The resulting matrix representing the morphology of a normal E17.5 individual who has followed the growth pattern specific to mice carrying an *Fgfr2* mutation, is subjected to a spectral decomposition (i.e., principal coordinates analysis) to obtain the 3D coordinates of landmark locations for the hypothetical form [29]. A check of the dimensionality of the landmark coordinates stipulates that the sum of the first three eigenvalues must collectively exceed 95% of the sum of all the eigenvalues validating the hypothetical form as biologically possible [24,29]. In our examples, the first three eigenvalues of the hypothetical forms constituted >99% of the sum of the eigenvalues. Hypothetical forms thus produced can be compared to age-matched samples or analyzed using ordination methods like PCA (Figure 7) to identify the groups to which they are most phenotypically similar. Because the simulated morphologies (Hypothetical forms A and B) and the unaffected mice share the same initial morphology at E17.5*,* differences between the hypothetical forms and unaffected P0 mice can be attributed exclusively to differences in growth pattern caused by the presence of an *Fgfr2* mutation.

### Suture analysis

All sutures were scored using isosurfaces segmented for bone using hydroxyapatite phantoms imaged with the specimens, as described above. In the case of the appearance of fusion, we examined individual HRμCT slice images for that portion of the suture to verify fusion. For each specimen, sutures were scored qualitatively as open (O) when more than 75% of the length of the suture was completely patent; partial (P) when more than 25% but less than 75% of the length of the suture was patent; and fused (F) when less than 25% of the length of the suture was patent.

## Competing interests

The authors declare they have no competing interests.

## Authors’ contributions

SMMP participated in the design of the study, carried out the morphometric analysis of form and growth, participated in the drafting of the manuscript, and made all reconstructions and visualizations. TMC performed the EDMA-based PCA analyses and participated in the design of additional analyses and interpretation of results; NM-A worked with original image data sets, contributed to statistical analysis and interpretation, and critically revised the manuscript; KA participated in the design of the study and critically revised the manuscript; EWJ participated in the design of the study and critically revised the manuscript; JTR conceived of the study, designed the analysis, coordinated the analyses and interpretation of results, and drafted the manuscript. All authors read, edited, and approved the final manuscript.

## Supplementary Material

Additional file 1: Table S1 Anatomical definitions of landmarks collected from HRμCT isosurfaces and used in analysis. Click here for file

Additional file 2: Table S2 Results (p-values) of nonparametric null hypothesis tests for form differences between *Fgfr2*^*+/S252W*^*and Fgfr2*^*+/P253R*^ mutant mice and their respective unaffected littermates at E17.5 and P0. Click here for file

Additional file 3 **Apert252_E17.5_EDMA.** Video 1: 3D reconstruction of isosurface of E17.5 mouse skull showing linear differences in cranial morphology as measured between 3D coordinates of anatomical landmarks between *Fgfr2*^*+/S252W*^ mice and unaffected littermates when landmarks are analyzed by EDMA, following Figure 4A and B. Blue lines are significantly larger in mutant mice relative to unaffected littermates. Fuchsia lines are significantly smaller in mutant mice. Bone segmented from HRμCT images is shown as partially transparent to better visualize the growth differences. Click here for file

Additional file 4 **Apert253_E17.5_EDMA.** Video 2: 3D reconstruction of isosurface of E17.5 mouse skull showing linear differences in cranial morphology as measured between 3D coordinates of anatomical landmarks between *Fgfr2*^*+/P253R*^ mice and unaffected littermates following Figure 4E and F. Blue lines are significantly larger in mutant mice relative to unaffected littermates. Fuchsia lines are significantly smaller in mutant mice. Bone segmented from HRμCT images is shown as partially transparent to better visualize the growth differences. Click here for file

Additional file 5 **Apert252_P0_EDMA.** Video 3: 3D reconstruction of isosurface of P0 mouse skull showing linear differences in cranial morphology as measured between 3D coordinates of anatomical landmarks between *Fgfr2*^*+/S252W*^ mice and unaffected littermates when landmarks are analyzed by EDMA, following Figure 4C and D. Blue lines are significantly larger in mutant mice relative to unaffected littermates. Fuchsia lines are significantly smaller in mutant mice. Bone segmented from HRμCT images is shown as partially transparent to better visualize growth differences. Click here for file

Additional file 6 **Apert253_P0_EDMA.mpg.** Video 4: 3D reconstruction of isosurface of P0 mouse skull showing linear differences in cranial morphology as measured between 3D coordinates of anatomical landmarks between *Fgfr2*^*+/P253R*^ mice and unaffected littermates following Figure 4G and H. Blue lines are significantly larger in mutant mice relative to unaffected littermates. Fuchsia lines are significantly smaller in mutant mice. Bone segmented from HRμCT images is shown as partially transparent to better visualize the growth differences. Click here for file

Additional file 7 **Table S3_GDMA_results.** Table S3. Results of null hypothesis testing for differences in growth between *Fgfr2*^*+/S252W*^*and Fgfr2*^*+/P253R*^ mutant mice and unaffected littermates. Click here for file

Additional file 8 **Apert252_GDMA.** Video 5: Differences in cranial growth between E17.5 and P0 as determined by GDMA of 3D landmarks of *Fgfr2*^*+/S252W*^ mice and unaffected littermates following Figure 6A and B. Linear distances that grew significantly more (blue) and significantly less (white) in mutant mice are shown on a 3D reconstruction of an isosurface of an E17.5 mouse skull. Thin lines indicate linear distances that showed growth magnitudes 5-10% different between mice carrying one of the two Fgfr2 mutations and their respective unaffected littermates; thick lines indicate linear distances that showed growth magnitudes >10% different between unaffected and mutant mice. Bone segmented from HRμCT images is shown as partially transparent to better visualize the growth differences. Click here for file

Additional file 9 **Apert253_GDMA.** Video 6: Differences in cranial growth between E17.5 and P0 as determined by GDMA of 3D landmarks of *Fgfr2*^*+/P253R*^ mice and unaffected littermates following Figure 6C and D. Linear distances that grew significantly more (blue) and significantly less (white) in mutant mice are shown on a 3D reconstruction of an isosurface of an E17.5 mouse skull. Thin lines indicate linear distances that showed growth magnitudes 5-10% different between mice carrying one of the two Fgfr2 mutations and their respective unaffected littermates; thick lines indicate linear distances that showed growth magnitudes >10% different between unaffected and mutant mice. Bone segmented from HRμCT images is shown as partially transparent to better visualize the growth differences. Click here for file
