# Supplementary material for: Craniofacial divergence by distinct prenatal growth patterns in Fgfr2 mutant mice
Source: BMC Dev Biol. 2014 Feb 28;14:8. doi: 10.1186/1471-213X-14-8 (PMC4101838; doi:10.1186/1471-213X-14-8)
Supplement: Additional file 1: Table S1 — Anatomical definitions of landmarks collected from HRμCT isosurfaces and used in analysis. [file 1471-213X-14-8-S1.docx]

Table S1. Anatomical definitions of landmarks collected from HRµCT isosurfaces and used in analysis. G: global skull, F: face, B: cranial base, V: cranial vault, P: palate. These landmarks and others may be viewed at: <http://getahead.psu.edu/landmarks_new.html>

| **Abbreviation** | **Anatomical definition** | **Skull region** |
| --- | --- | --- |
| lfbc, rfbc | Most lateral point of the frontal bone, taken along the basal part of the coronal suture, between the frontal and the parietal bones | G, V |
| lplp, rplp | Most posterolateral point on the parietal | G, V |
| lflac, rflac | Intersection of frontal process of maxilla with frontal and lacrimal bones | G, F |
| lzyt, rzyt | Intersection of zygoma with zygomatic process of temporal, taken on zygoma | G, F |
| laalf, raalf | Most anterior point of the anterior palatine foramen | G, F |
| lpalf, rpalf | Most posterior point of the anterior palatine foramen | G, F |
| lpmx, rpmx | Most inferolateral point of the premaxillarymaxillary suture, taken on premaxilla | G, F |
| rmaxi | Midline point on the premaxilla between the incisor and the nasal cavity just anterior of the incisive foramen, right side only | G, F |
| ethma | Most anterosuperior point of the vomer | G, F |
| ethmp | Most posterior point of the vomer | G |
| lasph, rasph | Posteromedial point of the inferior portion of the left alisphenoid | G, B |
| lpns, rpns | Most anterolateral indentation at the posterior edge of the horizontal plate of the palatine bone | G |
| lptyp, rptyp | Most posterior tip of the medial pterygoid process | G, B |
| lsyn, rsyn | Most anterolateral point on corner of the basioccipital at the basi occipital synchondrosis | G, B |
| bas | Midpoint on the posterior margin of the foramen magnum, taken on basioccipital | G, B |
| loci, roci | Superior posterior point on the ectocranial surface of occipital lateralis on the foramen magnum | G, B |
| lpsq, rpsq | Most posterior point on the posterior extension of the forming squamosal | G |
| lpto, rpto | Most posteromedial point on the parietal | G, V |
| amsph | Most anteromedial point on the body of the sphenoid | G, B |
| lalp, ralp | Most anterolateral point on the posterior palatine plate | P |
| ramp | Most anteriomedial point on the posterior palatine plate | P |
| lpmp | Most posteromedial point on the posterior palatine plate | P |
| lplpp, rplpp | Most posterolateral point on the posterior palatine plate | P |
