# Supplementary material for: Craniofacial divergence by distinct prenatal growth patterns in Fgfr2 mutant mice
Source: BMC Dev Biol. 2014 Feb 28;14:8. doi: 10.1186/1471-213X-14-8 (PMC4101838; doi:10.1186/1471-213X-14-8)
Supplement: Additional file 2: Table S2 — Results (p-values) of nonparametric null hypothesis tests for form differences between Fgfr2 +/S252W and Fgfr2 +/P253R mutant mice and their respective unaffected littermates at E17.5 and P0. [file 1471-213X-14-8-S2.docx]

Table S2. Results (p-values) of nonparametric null hypothesis tests for form differences between *Fgfr2^+/S252W^ and Fgfr2^+/P253R^* mutant mice and their respective unaffected littermates at E17.5 and P0. A p-value of 0.05 or less was considered statistically significant enabling rejection of the null hypothesis of similarity in shape. Landmark subsets for anatomical regions are defined in Table S1.

|  | ***Fgfr2^+/S252W^*** | | ***Fgfr2^+/P253R^*** | |
| --- | --- | --- | --- | --- |
| Landmark subset | **E17.5** | **P0** | **E17.5** | **P0** |
| Global skull | 0.017 | 0.001 | 0.002 | 0.001 |
| Cranial base | 0.001 | 0.001 | 0.002 | 0.001 |
| Facial skeleton | 0.001 | 0.001 | 0.025 | 0.001 |
| Cranial Vault | 0.122 | 0.001 | 0.029 | 0.001 |
| Palate | 0.012 | 0.001 | 0.78 | 0.02 |
