# Supplementary material for: Craniofacial divergence by distinct prenatal growth patterns in Fgfr2 mutant mice
Source: BMC Dev Biol. 2014 Feb 28;14:8. doi: 10.1186/1471-213X-14-8 (PMC4101838; doi:10.1186/1471-213X-14-8)
Supplement: Additional file 7 — Table S3_GDMA_results. Table S3. Results of null hypothesis testing for differences in growth between Fgfr2 +/S252W and Fgfr2 +/P253R mutant mice and unaffected littermates. [file 1471-213X-14-8-S7.docx]

Table S3. Results of null hypothesis testing for differences in growth between *Fgfr2^+/S252W^ and Fgfr2^+/P253R^* mutant mice and unaffected littermates. A p-value of 0.05 or less was considered statistically significant enabling rejection of the null hypothesis of similarity in growth pattern between samples. Landmark subsets for anatomical regions are defined in Table S1.

|  | Growth of *Fgfr2^+/S252W^* compared to growth of unaffected littermates | Growth of *Fgfr2^+/P253R^*  compared to growth of unaffected littermates | Growth of *Fgfr2^+/P253R^* compared with growth of *Fgfr2^+/S252W^* |
| --- | --- | --- | --- |
| Landmark subset | P-Value | P-Value | P-value |
| Global skull | 0.092 | 0.053 | 0.021 |
| Cranial base | 0.022 | 0.179 | 0.668 |
| Facial skeleton | 0.001 | 0.004 | 0.154 |
| Cranial Vault | 0.021 | 0.095 | 0.114 |
| Palate | 0.012 | 0.012 | 0.107 |
